# Introduction: Organ Specific Immunopathology

**DOI:** 10.1080/17402520412331298042

**Published:** 2004

**Authors:** M. Eric Gershwin, Yehuda Shoenfeld


*Clinical & Developmental Immunology*, September/December 2004, Vol. 11 (3/4), p. 189

Taylor & Francis healthsciences

*Introduction*

### Organ Specific Immunopathology

The first spontaneous animal model of autoimmune disease was discovered in New Zealand in 1959 and appropriately named, New Zealand Mice. The influence of the immune system on health is now known to go far beyond that of protection from infectious disease and the converse immunological attack on self. The influence of immunity can be seen in diseases as far reaching as success in wound healing to the inflammatory process following myocardial infarction. In this special issue, we include a large number of papers that are presented at the 4th International Congress on Autoimmunity in Budapest, Hungary, November 3–7, 2004. We are also including several others that were not presented because they highlight the focus on the interesting spectrum of the role of the immune response.

Indeed, it was not until the modern era of medicine that the role of immunity, immune pathology and allergy, was even appreciated. In fact, it was not until after 1950 that a critical nucleus of researchers interested in autoimmunity appeared. One of the earliest descriptions of immune mediated pathology was that of hemolytic anemia, described by Donath and Landsteiner in 1904. Clearly, several key landmarks occurred thereafter, including the description of rheumatoid factor in 1941, the direct Coombs test in 1945, and the LE cell test in 1948. The first clear description of the wide range of autoimmune diseases was the classic text of Mackay and Burnet entitled Autoimmune Diseases (Pathogenesis, Chemistry and Therapy). We note that we are honoring Dr Ian Mackay at the Budapest Meeting for his lifetime achievement in immunology and, in particular, in autoimmune disease. The papers included in this issue focus on the organ specific immunopathology, including diseases such as primary biliary cirrhosis, myasthenia gravis, autoimmune diabetes, and multiple sclerosis. We also include interesting issues of the role of the immune system in wound healing as well as the increasing evidence for the influence of the immune response on both pregnancy loss and endometriosis. With the evolution of our journal, Clinical and Developmental Immunology, with its increasing interest in immune-mediated pathology, we thought this collection of papers would be particularly appropriate. The collection is certainly not meant to be complete, but rather to represent the exciting spectrum of the immune response.

M. Eric Gershwin, M.D.
Yehuda Shoenfeld, M.D.

ISSN 1740-2522 print/ISSN 1740-2530 online © 2004 Taylor & Francis Ltd
DOI: 10.1080/17402520412331298042

